# Revealing an Uncommon Presentation of Chiari I Malformation With Diverse Craniovertebral Anomalies in the Absence of Syringomyelia and Atlanto-Occipital Subluxation: A Case Report

**DOI:** 10.7759/cureus.55332

**Published:** 2024-03-01

**Authors:** Nabha Mahajan, Suresh Phatak, Prashant Onkar, Ashish N Ambhore, Pranit Pantawane

**Affiliations:** 1 Radiodiagnosis, Narendra Kumar Prasadrao (NKP) Salve Institute of Medical Sciences and Research Centre, Nagpur, IND; 2 Radiodiagnosis, Datta Meghe Institute of Medical Sciences, Wardha, IND

**Keywords:** chiari i malformation, cvj, klippel-feil syndrome, basilar invagination, platybasia, craniovertebral junction anomalies

## Abstract

A Chiari I malformation is a frequently encountered anomaly of the posterior fossa, occurring in a notable percentage of the population. It often coexists with various other craniovertebral junction abnormalities, albeit less frequently with Klippel-Feil syndrome. Interestingly, the majority of individuals with Chiari I malformation do not exhibit any symptoms. We present a rare case of a 25-year-old male with chronic neck and occipital pain, along with progressive weakness and sensory disturbances in all four limbs, urinary urgency, and elevated left shoulder. Examination unveiled spasticity, weakness, and brisk reflexes. On extensive radiological evaluation (X-ray, CT, and MRI), findings revealed various anomalies in the craniovertebral junction, including complete atlanto-occipital assimilation, basilar invagination, and platybasia. Furthermore, cervical segmentation abnormalities indicative of Klippel-Feil syndrome were observed, along with Sprengel's deformity. MRI confirmed Chiari I malformation with tonsillar herniation and myelomalacia, as well as compression at the cervico-medullary junction. This patient underwent a surgical procedure that included transoral odontoidectomy combined with occipito-cervical fixation, after which a good clinical response was observed. It emphasizes the necessity of radiological imaging for the diagnosis of Chiari and other associated abnormalities in the craniovertebral junction.

## Introduction

The craniovertebral junction (CVJ) serves as the crucial interface between the spine and the skull, comprising various bony and soft tissue structures [1]. Anomalies in this region can lead to diverse clinical manifestations, from headaches to neurological deficits, necessitating a thorough understanding and precise radiological assessment [2].

Utilizing advanced imaging techniques like CT and MRI, radiologists can accurately evaluate CVJ abnormalities such as Chiari I malformation, characterized by cerebellar tonsillar descent into the upper cervical canal [3]. Basilar invagination, involving upward displacement of the foramen magnum margin [4], and Klippel-Feil syndrome, stemming from defective spine segmentation, are also prevalent CVJ anomalies [5].

Additionally, associations like syringomyelia with Chiari I malformation underscore the complexity of CVJ pathology and highlight the need for comprehensive evaluation and management tailored to each patient's unique presentation [6].

This particular case is unique due to its absence of syringomyelia and atlantoaxial subluxation, contrasting with common associations observed in Chiari I malformation cases. The absence of these typical features highlights the individuality of this case and underscores the importance of a comprehensive assessment and management of CVJ anomalies.

## Case presentation

**Figure 1:**
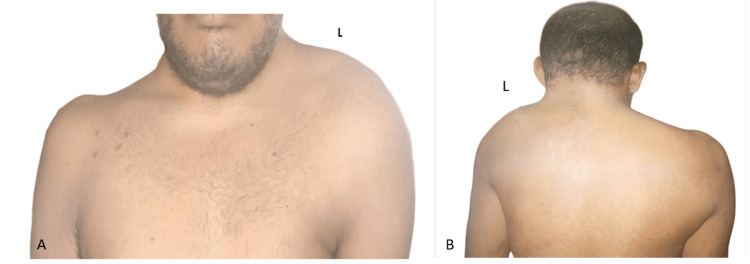
A) Ventral view. B) Dorsal view. The left shoulder was elevated as compared to the right side.

A 25-year-old male presented to us with a one-year history of neck and occipital pain and progressive weakness in all four limbs. He also had paresthesias and numbness in the upper limbs, urinary urgency, and precipitancy for six months. On examination, spasticity was present in both upper and lower limbs. Power was 4/5 across all the joints and the hand grip strength was weak. Reflexes were all brisk. Left shoulder was elevated as compared to right side. (Figures 1A, 1B).

Based on the symptomatology, a probable localization to cervical cord was thought of and further investigations were undertaken to look for the etiology.

**Figure 2:**
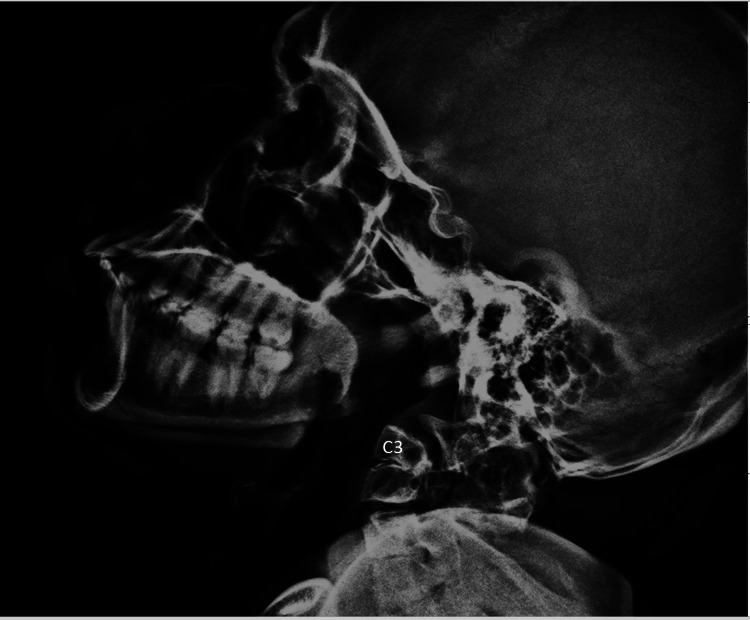
On lateral X-ray imaging, assimilation of the anterior arch with occipital bone and protrusion of odontoid process in the posterior fossa was noted. C3-C4 vertebral bodies appeared partially fused.

Routine blood investigations were normal. The radiological findings revealed several anomalies in the cervical spine. On lateral X-ray imaging (Figure 2), assimilation of the anterior arch with occipital bone and protrusion of dens in the posterior fossa was visualized. C3-C4 vertebral bodies were partially fused. The patient was recommended to undergo CT imaging.

**Figure 3:**
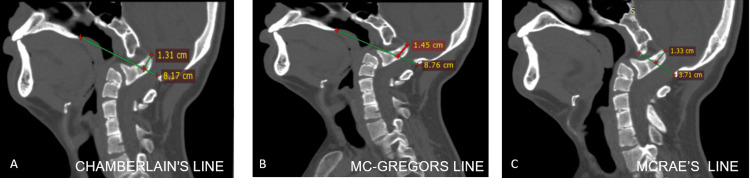
CT sagittal reformatted image demonstrated (A) the tip of the odontoid was 13.1 mm above Chamberlain's line, (B) the tip of the odontoid was 14.5mm above McGregor's line, and (C) the tip of the odontoid was positioned 13.3 mm above McRae's line. The above features suggested a diagnosis of basilar invagination.

**Figure 4:**
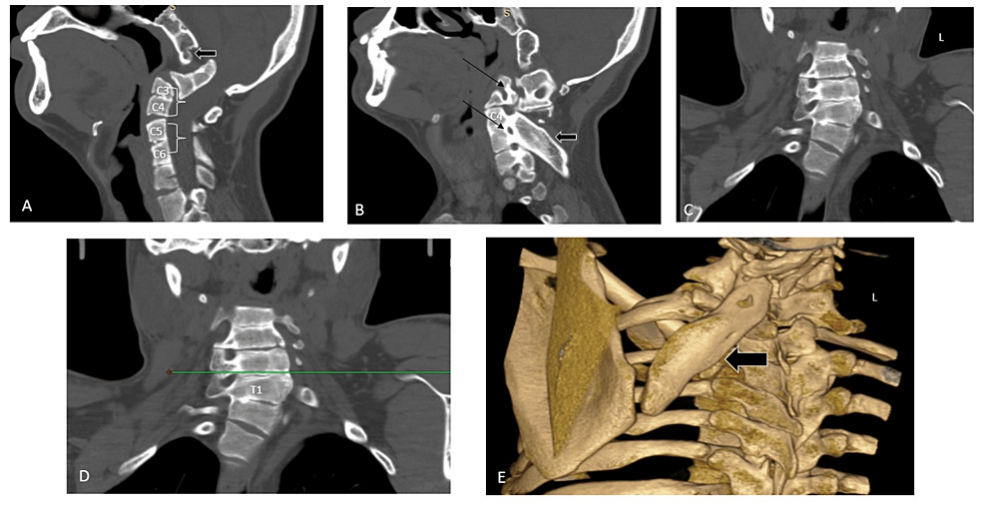
A) CT sagittal reformatted image demonstrated a complete atlanto-occipital assimilation (black arrow), a partial fusion of C3-C4 and C5-6 vertebral bodies, and retrolisthesis of the C4 over the C5 vertebra. B) CT sagittal reformatted image illustrated the fusion of the posterior elements of C3-C4 and C5-C6 (thin arrows), omovertebral bone (broad black arrow) connecting the cervical spine (C5-6 transverse process) to the scapula. C) CT coronal reformatted image demonstrated scoliosis with convexity toward the left side. D) Elevated scapula with superomedial angle (line) of scapula at T1 vertebral body suggestive of Sprengel's deformity (Grade II). E) CT 3D reconstructed image showed the omovertebral bone (black arrow) connecting the cervical spine and the superomedial border of the scapula.

On CT imaging of the CVJ, the X-ray findings were confirmed, which included basilar invagination (the tip of dens was 13.1 mm over Chamberlain's line (Figure 3A), 14.5 mm over McGregor's line (Figure 3B) and 13.3 mm over McRae's line (Figure 3C)), complete atlanto-occipital assimilation along with various cervical segmentation abnormalities including a partial fusion of C3-C4, C5-6 vertebral bodies, retrolisthesis of the C4 over the C5 vertebra (Figure 4A), and the fusion of the posterior elements of C3-C4 and C5-C6 (Figure 4B). Additionally, the presence of the right cervical rib and the bifid sternal end of the left first rib was also noted.

On coronal reformatted images, scoliosis with leftward convexity was noted (Figure 4C). The scapula was raised, with the superomedial angle of the scapula aligned at the level of the T1 vertebral body suggestive of Grade II Sprengel's deformity (Rigault’s classification) (Figure 4D). An omovertebral bone connecting the superomedial scapular border to the cervical spine (C5-6 transverse process) was also visualized (Figures 4B, 4E).

**Figure 5:**
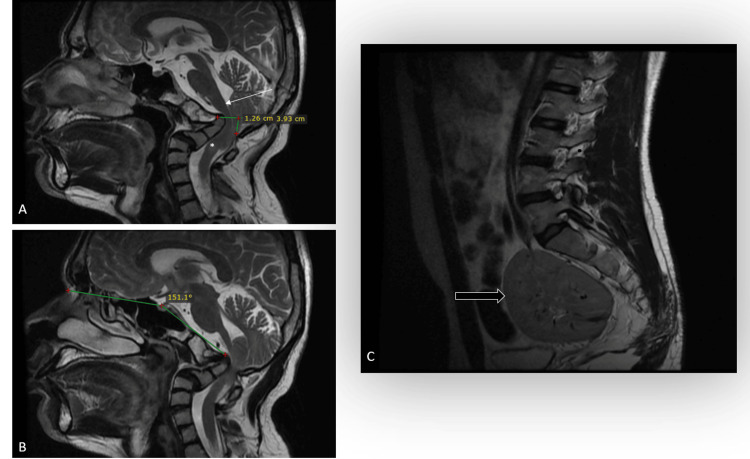
A) MRI cervical spine sagittal imaging illustrated that the cerebellar tonsils were displaced inferiorly through the foramen magnum for a length of 12.6 mm, associated with changes of myelomalacia (asterisk) at the level of the C2 vertebral body. It also showed significant compression of cervicomedullary junction as the dens invaginate the posterior fossa (arrow). B) MRI cervical spine sagittal imaging demonstrated the Welcher basal angle measuring 151 degrees, suggestive of platybasia. C) MRI sagittal spine screening demonstrated a single pelvic kidney (arrow).

MRI imaging of the cervical spine was conducted to evaluate the spinal cord involvement. The T2-weighted sagittal image revealed peg-shaped cerebellar tonsils with a downward displacement of 12.6 mm, indicative of tonsillar herniation (Figure 5A). An altered signal intensity area was detected at the C2 vertebral body level, exhibiting hyperintense on T2-weighted imaging and hypointense on T1-weighted imaging, suggestive of myelomalacia. The imaging also demonstrated significant compression at the cervicomedullary junction resulting from dens invagination into the posterior fossa (Figure 5A).

The Welcher basal angle measured 151 degrees, which was suggestive of platybasia (Figure 5B). An incidental finding of a single pelvic kidney was also noted on sagittal MRI spine screening (Figure 5C). The cardiac evaluation was normal.

Radiological findings confirmed Chiari I malformation with multiple craniovertebral abnormalities, including platybasia, basilar invagination, and Klippel-Feil syndrome with Sprengel's deformity. The patient underwent transoral odontoidectomy, resection of the omovertebral bone, and occipitocervical fixation, following which there was a significant improvement. He was advised to continue physiotherapy at discharge. At a follow-up after six months, the patient was independent in all activities of daily living (ADLs).

## Discussion

The CVJ is the transitional region connecting the spine and the skull [1]. It consists of bony structures (occipital bone, atlas and axis vertebrae) connected by synovial joints, along with intrinsic ligaments, membranes, and muscles [4]. The CVJ is formed by the fusion of four occipital somites. The fourth occipital somite fuses with the cranial segment of the first cervical somite, forming proatlas sclerotome, a precursor to the CVJ. The formation of the CVJ from this precursor takes place during the gestational period from week four to week 12 [1].

The CVJ exhibits a variety of bony abnormalities [4]. The clinical manifestations vary, including chronic headaches, restricted neck motion, and neurological deficits [2]. The CVJ anomalies are frequently linked with conditions such as Chiari malformation, Klippel-Feil syndrome, and syringomyelia [4,6]. Patients with symptomatic abnormalities in the CVJ have a 33-38% chance of having accompanying hindbrain abnormalities [6]. They are more common in males and predominantly in the age group of 11-20 years [7]. A thorough understanding of these abnormalities is essential for preoperative planning [4,6]. The assessment of the CVJ requires the identification of specific normal landmarks [4]. It’s crucial to obtain an image of the skull or brain capturing key landmarks like the hard palate, basion, and tuberculum sella [4]. The measurement on plain radiographs is often challenging due to the superimposition of bony structures [4]. CT gives superior visualization of the osseous structures. MRI additionally gives information regarding the surrounding soft-tissue structures around the CVJ [2].

The Chiari I malformation involves the descent of the cerebellar tonsils by a minimum of 5 mm into the upper cervical canal [3]. Various pathogenic mechanisms have been postulated [3]. The Chiari malformation is linked to additional CVJ anomalies up to 74% [8], with less than 5% being associated with Klippel-Feil syndrome [3,9].

Basilar invagination involves the upward displacement of the margin of the foramen magnum and upper cervical spine extending into the base of the skull [4]. For its diagnosis on radiological imaging, the tip of the dens protrudes more than 7 mm above McGregor's basal line or 5 mm above Chamberlain's line [4]. The primary form is congenital, while secondary basilar invagination, or basilar impression, is associated with skull base softening due to an acquired disease [2].

Platybasia and basilar invagination frequently manifest in conjunction with various disorders including Chiari malformations [10]. A revised angle has been introduced for MRI assessment, with the two tangents subtending at the dorsum sella instead of the center of the sella [11]. The modification results in a decreased angle compared to standard craniometric measurements (ranging from a minimum of 100° to a maximum of 127°) [11]. It is crucial to be mindful of the specific landmarks utilized to derive the reported angle [4].

Occipitalization of the atlas involves a congenital fusion between the occipital bone and the atlas. The occurrence varies from 0.08% to 3% in the general population, varying from a fibrous connection connecting the occiput to a small region of the atlas or complete fusion to a bony bridge [12].

Klippel-Feil syndrome arises from a defect in spine segmentation during embryonic development. This results in a spine lacking intervertebral disc and cartilage, thus impacting overall instability [5]. Beyond cervical segmentation anomalies, various CVJ anomalies and visceral and congenital anomalies are also observed [5,7]. Rigault proposed a classification system for evaluating the Sprengel deformity and identifying the presence of omovertebral bone communication by analyzing the relationship of the superomedial angle to the vertebral column. [13].

Syringomyelia is prevalent, occurring in 40-75% of Chiari I malformation cases. It frequently develops in individuals with significant tonsillar herniation and obstruction of CSF flow, typically between the C4 and C6 levels [14]. The documented correlation between Chiari I malformation and atlantoaxial subluxation is approximately 30% [15].

Therefore, we deduce associations of various anomalies with Chiari I malformation, as observed in this particular case. Notably, our case stands out due to the absence of syringomyelia and atlantoaxial subluxation.

## Conclusions

The CVJ, a distinctive element of the ligamento-osseous craniospinal axis, possesses unique embryological origins and specific biomechanical requirements. Diagnosing complexities in this region poses a challenge for imaging specialists. While plain radiographs play a crucial role initially, detailed insights into bony and soft tissue structures are optimally obtained through multiplanar imaging using CT and MRI. A profound grasp of the distinctive pathology and anatomy from an imaging viewpoint is crucial for effective operative planning.
